# TAILR (Nursing-Sensitive Events and Their Association With Individual Nurse Staffing Levels) Project: Protocol for an International Longitudinal Multicenter Study

**DOI:** 10.2196/56262

**Published:** 2024-04-22

**Authors:** Stefanie Bachnick, Maria Unbeck, Maryam Ahmadi Shad, Katja Falta, Nicole Grossmann, Daniela Holle, Jana Bartakova, Sarah N Musy, Sarah Hellberg, Pernilla Dillner, Fatemeh Atoof, Mohammadhossein Khorasanizadeh, Paula Kelly-Pettersson, Michael Simon

**Affiliations:** 1 Department of Nursing Science University of Applied Sciences Bochum Germany; 2 School of Health and Welfare Dalarna University Falun Sweden; 3 Department of Clinical Sciences Danderyd Hospital Karolinska Institutet Stockholm Sweden; 4 Institute of Nursing Science Department Public Health, Faculty of Medicine University of Basel Basel Switzerland; 5 Health Economics Facility Department of Public Health University of Basel Basel Switzerland; 6 Department of Orthopaedics Danderyd University Hospital Stockholm Sweden; 7 Department of Women's and Children's Health Karolinska Institutet Stockholm Sweden; 8 Department of Neonatology Astrid Lindgren Children's Hospital Karolinska University Hospital Stockholm Sweden; 9 Social Determinants of Health Research Center Kashan University of Medical Sciences Kashan Iran; 10 Trauma Nursing Research Center Kashan University of Medical Sciences Kashan Iran

**Keywords:** adverse events, electronic health record, hospital care, no-harm incidents, nursing care, nursing-sensitive events, nurse staffing, patient safety, systematic record review

## Abstract

**Background:**

Nursing-sensitive events (NSEs) are common, accounting for up to 77% of adverse events in hospitalized patients (eg, fall-related harm, pressure ulcers, and health care–associated infections). NSEs lead to adverse patient outcomes and impose an economic burden on hospitals due to increased medical costs through a prolonged hospital stay and additional medical procedures. To reduce NSEs and ensure high-quality nursing care, appropriate nurse staffing levels are needed. Although the link between nurse staffing and NSEs has been described in many studies, appropriate nurse staffing levels are lacking. Existing studies describe constant staffing exposure at the unit or hospital level without assessing patient-level exposure to nurse staffing during the hospital stay. Few studies have assessed nurse staffing and patient outcomes using a single-center longitudinal design, with limited generalizability. There is a need for multicenter longitudinal studies with improved potential for generalizing the association between individual nurse staffing levels and NSEs.

**Objective:**

This study aimed (1) to determine the prevalence, preventability, type, and severity of NSEs; (2) to describe individual patient-level nurse staffing exposure across hospitals; (3) to assess the effect of nurse staffing on NSEs in patients; and (4) to identify thresholds of safe nurse staffing levels and test them against NSEs in hospitalized patients.

**Methods:**

This international multicenter study uses a longitudinal and observational research design; it involves 4 countries (Switzerland, Sweden, Germany, and Iran), with participation from 14 hospitals and 61 medical, surgery, and mixed units. The 16-week observation period will collect NSEs using systematic retrospective record reviews. A total of 3680 patient admissions will be reviewed, with 60 randomly selected admissions per unit. To be included, patients must have been hospitalized for at least 48 hours. Nurse staffing data (ie, the number of nurses and their education level) will be collected daily for each shift to assess the association between NSEs and individual nurse staffing levels. Additionally, hospital data (ie, type, teaching status, and ownership) and unit data (ie, service line and number of beds) will be collected.

**Results:**

As of January 2024, the verification process for the plausibility and comprehensibility of patients’ and nurse staffing data is underway across all 4 countries. Data analyses are planned to be completed by spring 2024, with the first results expected to be published in late 2024.

**Conclusions:**

This study will provide comprehensive information on NSEs, including their prevalence, preventability, type, and severity, across countries. Moreover, it seeks to enhance understanding of NSE mechanisms and the potential impact of nurse staffing on these events. We will evaluate within- and between-hospital variability to identify productive strategies to ensure safe nurse staffing levels, thereby reducing NSEs in hospitalized patients. The TAILR (Nursing-Sensitive Events and Their Association With Individual Nurse Staffing Levels) study will focus on the optimization of scarce staffing resources.

**International Registered Report Identifier (IRRID):**

DERR1-10.2196/56262

## Introduction

### Overview

The link between patient outcomes and adequate nurse staffing levels has been extensively studied internationally over the last 2 decades. To avoid negative patient outcomes, such as adverse events, adequate staffing levels and combinations of skills and grades are needed. In hospital settings, up to 77% of adverse events are attributed to nursing care [1]. Nursing-sensitive events (NSEs) are part of adverse events that are specifically affected, caused, and influenced by the processes or structures of nursing care, though nursing is not exclusively responsible [2]. Examples of NSEs are hospital-acquired urinary tract infections, pressure ulcers, pneumonia, or deep venous thrombosis [3]. NSEs may be accompanied by additional monitoring or treatment [4]. Compared to studies on adverse events and their characteristics, few studies include all types of NSEs and their prevalence, preventability, and severity [5]. A recently published study exploring NSEs in home care settings found that 73% of the NSEs were preventable, and approximately 37% resulted in temporary harm that required additional health care resources [6]. In addition to the burden on the patient, NSEs generate high medical costs by necessitating additional monitoring or treatment [5,7]. Different methods to identify and assess NSEs are available. Currently, there is no gold standard for identifying and assessing NSEs. However, retrospective record review (RRR) methods, such as the Global Trigger Tool [5] and the Harvard Medical Practice Study methodology [8], seem most promising. Compared to other methods (eg, critical incident reporting systems and patient safety indicators), record review facilitates the detection of more adverse events, thereby minimizing the underreporting of NSEs [9]. To prevent and reduce NSEs and ensure quality nursing care, appropriate nurse staffing levels are needed [8,10]. Nurse staffing refers to the number and qualifications of nurses relative to the care demands of patients, often expressed as staffing levels (eg, nurse-to-patient ratios), skill mix (ie, the composition of the nursing team in terms of qualifications), and grade mix (ie, educational levels of the nursing team) [9]. Providing optimal nurse staffing levels has been a challenge for health care systems worldwide. Having too few nurses increases the risk of NSEs and decreases the quality of care (F Gratwohl, N Grossmann, S Musy, and M Simon, unpublished data, September 2018) [11,12]; it also increases the risk of nurse burnout and job dissatisfaction, which impedes the recruitment and maintenance of the nursing workforce [13]. Evidence suggests that the occurrence of negative patient outcomes is related to staffing [14], but there is a lack of systematic reviews focusing on different types of NSE.

Despite studies that substantiated the link between nurse staffing and NSEs [14-16], operational and methodological challenges have limited the practical impact of this research. Typically, staffing studies describe only constant staffing exposure at the unit or hospital level without assessing individual patient-level exposure to nurse staffing during the hospital stay [17]. Methodological challenges include the omission of important variables (eg, shift patterns, skill mix, and patient turnover) [10,16]; common method variance (ie, dependent and independent variables derived from the same survey of nurses); and simultaneity, which refers to common-cause variables affecting both staffing and outcomes, such as patient severity [16]. Only a few studies have assessed nurse staffing and patient outcomes using a longitudinal study design [10,15,18], and most of these studies were single-center studies; there is a lack of multicenter or international studies allowing generalizability. Therefore, there is a need for multicenter longitudinal studies of the association between individual patient-level exposure to nurse staffing and all types of NSEs, with improved potential for generalization.

To advance the field, there is a strong need to determine the optimal intervention strategies for nurse staffing levels required to reduce the risk of NSEs. This study is the first step in generating data aimed at structuring and planning future intervention strategies.

### Objectives

The overall aim of the TAILR (Nursing-Sensitive Events and Their Association With Individual Nurse Staffing Levels) study is to investigate the association between NSEs and individual nurse staffing levels. The specific aims are as follows:

1. To determine the prevalence, preventability, type, and severity of NSEs across sites using structured RRR methodology
2. To describe individual patient-level nurse staffing across hospitals
3. To describe the effect of nurse staffing on NSEs in hospitalized patients
4. To determine thresholds of safe nurse staffing levels and test them against NSEs in hospitalized patients

## Methods

### Design

TAILR is an international multicenter study with a longitudinal and observational research design, running from 2021 to 2025. Altogether, 4 countries are participating in the study: Switzerland, Sweden, Germany, and Iran. Those countries make up the international TAILR consortium. Except for Switzerland, all data will be collected within the TAILR study. For Switzerland, data from the CroWiS (Crowd Working in der Schweiz) study will be used for data collection and analyses. CroWiS is a multimethod study with the overall aim of investigating the effect of temporary nurses in Switzerland [19].

### Setting and Sample

For the different countries, different numbers of hospitals and units are included in the TAILR study. The numbers of participating hospitals and units range from 1 to 4 hospitals and 4 to 21 units, depending on the country. Across all countries, 4 medical, surgical, or mixed units for each hospital are participating in the study, which is a total of 11 hospitals and 49 units. The study also includes acute care hospitals providing elective care. The inclusion criteria for units are as follows: they must have at least 10 patient beds and are either medical or surgical. Units with elevated levels of care, such as intensive or intermediate care, will be excluded, as these units have higher levels of staffing. In Sweden, 1 of the 4 hospitals is a pediatric hospital that is part of a university hospital providing adult care as well. For this pediatric hospital in the TAILR study, 9 units will be included. For pediatric care, neonatal care units will be included, but these data will be analyzed separately in the pediatric cohort. Table 1 shows the setting and sample information for each country.

During the 16-week observation period, 60 admissions will be randomly selected from all eligible admissions per unit and will be reviewed. For adult care, patients ≥18 years of age will be included. For pediatric care, no inclusion criteria for age will be applied. All patients must be hospitalized for at least 48 hours at a TAILR unit, and the admission record must be closed. The stay in the TAILR unit ends after patients have been discharged, when the length of stay exceeds 14 days or when patients are transferred from the TAILR unit for more than 12 hours. If the patient has a length of stay >14 days, the data collection ends at the end of day 14. If a patient is readmitted, this admission is considered a new admission.

**Table 1:** Number of hospitals, units, and patients, along with shift system information, including the number of shifts for each country and in total.

Country and hospital			Hospital, n		Units, n		Shifts per day, n		Patient admission, n		Number of shifts, n		
											Weekday		Weekend
**Germany**													
	Acute adult care hospital	3		12		3-14		720		Minimum 2880		Minimum 1152	
**Iran**													
	Acute adult care hospital	1		4		3		240		1152		192	
**Sweden**													
	Acute adult care hospital	3		12		3		720		2880		1152	
	Pediatric care hospital	1		9		3		560		2160		864	
	Total	4		21		N/A^a^		1280		5040		2016	
**Switzerland**													
	Acute adult care hospital	3		12		3		720		2880		1152	
**All countries**													
	Acute adult care hospital	10		40		N/A		2400		Minimum 9792		Minimum 3648	
	Pediatric care	1		9		N/A		560		2160		864	
	Total	11		49		N/A		2960		Minimum 11,952		Minimum 4512	

^a^N/A: not applicable.

### Variables and Measurements

#### Patient Characteristics and NSEs

Patient characteristics (eg, age and sex), clinically relevant variables (eg, primary diagnosis and patient-based workload for activities of daily living), and NSEs will be collected from patient records using systematic RRR, inspired by commonly used RRR methods. For the collection of the type of NSEs, we will use a set of predefined events (eg, deficiencies in drug management, deterioration of vital signs, health care–associated infections, gastrointestinal impairment, pain, and pressure ulcer) based on a literature review [3,5,6,20]. Furthermore, there is an option to enter “other” NSEs in the patient record, which allows all types of NSEs to be included. In addition to the type of NSE, preventability, severity, timing, origin, and potential contributing causes will be assessed per NSE. For severity, a slightly modified version of the National Coordinating Council for Medication Error Reporting and Prevention will be used, ranging from “An event that reached the patient but did not cause harm” to “Contributed or resulted in the patient’s death.”

#### Staffing Assessment

The shift-level nurse staffing data of the participating units will be collected using routine data or data from unit schedules depending on the hospital. For each day and each shift during the observation period of 16 weeks, each registered nurse (RN), specialist RN, nurse with an associate nursing degree (AND), unqualified helper, and student or trainee directly involved in patient care on the unit will be considered in the staffing assessment. Informal caregivers will be excluded. The assessment also includes whether the staff member is a temporary worker on the unit. We define “temporary” as a staff member deployed from a different unit than the target TAILR unit, a hospital pool, or an external agency. We will not differentiate between temporary nurses who only work for a single or few shifts and nurses who are deployed for weeks or months. To identify shifts with missing nurses, we will clarify whether the number of staff on this shift was as planned or not. If not, the missing number of nurses stratified by nursing degree will be entered.

#### Patient Counts and Turnovers

To assess the workload of nurses in relation to patient turnover, routine data regarding the number of patients, discharges, admissions, and transfers will be collected. Moreover, we will assess the number of out-of-specialty patients to account for additional workload for nurses.

#### Unit Survey

The study-specific 26-item unit survey assesses the organizational characteristics of the participating units, such as size (bed count) and service line. Additionally, data about the used care system and the used system to describe nursing care demands, workload, or patient acuity will be part of the unit questionnaire.

#### Hospital Survey

The study-specific hospital survey (8 items) will be used to collect hospital-level characteristics and statistics, such as ownership status (eg, private, not-for-profit, and public) and type (ie, district, general, and teaching hospital), as well as regulation, financing, and provision of the hospital.

#### Survey Translation and Validity Testing

The study manual, surveys, and assessments were developed in English. For Germany and Iran, they were translated into German and Farsi (Persian), respectively. Using a modified Brislin protocol, a systematic translation process was conducted [21]. After translation, an expert panel review of bilingual clinical and research nurses fluent in each target language reviewed each item in terms of cultural adaptations. To ensure comprehensibility and to check for response patterns, the entire German and Farsi versions of the NSE assessment sheet were pilot tested with nurses of varying educational levels. For all language versions, adaptations were made, as necessary, for wording and clarity.

#### Data Collection

The data observation period started in 2022. The data collection covers an observation period of 16 weeks at each hospital, but the start dates for hospital enrollment will be staggered between and within countries. We opted for this stepwise approach to ensure proper supervision and optimal use of resources during the data collection process, considering unit availability. Data will be collected digitally or with paper and pencil.

In Sweden and Switzerland, the random sampling of 60 admissions per unit will be carried out at the end of the 16-week observation period. In Germany and Iran, to use the available data collection resources, a random sampling of 15 admissions for each unit occurred 4 times with an interval of 4 weeks. Therefore, the number of included admissions in Germany and Iran is the same as those in the other participating countries, that is, 60 admissions per unit within 16 weeks of observation. Data will be collected by the internal RNs of the corresponding hospital or RNs who have experience working in the corresponding hospital. All data collectors will receive standardized education with the study manual, PowerPoint presentations, test examples, test records, and discussion sessions. For data quality control, an RN experienced in research for comprehensibility and plausibility will monitor the entered data.

The shift-level staffing assessments will be collected every day (from Monday to Sunday) for every shift (ie, morning, afternoon, night, and intermediated shifts). In Germany and Iran, data will be entered directly into a cloud-based electronic data capture platform by the ward manager of the TAILR unit, a hospital internal RN or a research team member. All data collectors for staffing assessment will receive a 3-hour education session with test entries and discussion sessions. In Sweden, shift data will be generated automatically by the corresponding hospital staffing system. In Switzerland, staffing data will be manually extracted from hospital staffing systems and entered into a secure web platform by members of the research team.

The hospital- and unit-level surveys will be conducted retrospectively after the main data collection. Data will be collected using routine data. If no routine data are available, interviews between TAILR country-specific research group members, unit managers, and hospital managers will be conducted.

Data will be stored in a cloud-based database, with a server in the Netherlands, that complies with the European Union General Data Protection Regulation. Access to the platform is restricted to the TAILR research group members and data collectors at specific sites.

#### Sample Size

We used 3 hospitals as the basis for sample size considerations. Assuming the same NSE rate of 36.4% as in a Swedish study [5], a sample size of 720 (95% CI 33.3%-39.5%) patients is expected (aim 1). A simulation (500 iterations per cell) of a 3-level generalized linear mixed model with a count variable for each shift above a low staffing threshold, with 15% of patients exposed to 1 or more shifts, was performed. We set the random effects at the unit and hospital level to 0.1, indicating an intraclass correlation coefficient (ICC) of 0.03. Based on an odds ratio of 1.10 and a sample of 60 patients per 4 units in 3 hospitals, the power was 100%. With a lower odds ratio of 1.07, representing the smallest effect of interest for a sample of 60 patients per 4 units in 3 hospitals, the power was still 0.91. As the ICC is unknown, we also assessed ICCs of 0, 0.06, and 0.15, resulting in the power ranging from 90% to 100% for both effect sizes, with a sample of 720 patients. Reducing the number of units to 3 per site, the total sample size was reduced to 540, with the power dropping to 70%-96%, depending on the specification. As the weights will only be known after the study has been conducted [22], this power calculation is based on a generalized linear mixed model without inverse probability weighting.

### Data Analysis

#### Aim 1

A descriptive analysis of the prevalence, preventability, types, and severity of NSEs will be carried out. We will calculate the frequencies and percentages with 95% CIs for NSE type, severity, and preventability. We will calculate the prevalence of NSEs per 1000 patient days and 100 patient admissions. To determine the quality of the review process, we will assess interrater reliability between 2 primary reviewers from a random sample of every tenth of the admissions via the Cohen kappa (κ) coefficient for review stage 1. Agreement between reviewers is defined as (1) agreement on the presence of potential NSEs by calculating Cohen kappa and (2) agreement on the number of NSEs by calculating the weighted Cohen kappa, where high disagreement corresponds to high weights.

#### Aim 2

In the first step, a descriptive analysis of the numbers of patients and nurses and the patient-to-nurse ratios for different staff categories (eg, RNs, specialist RNs, nurses with ANDs, and unqualified staff) over time will be conducted. Extreme staffing shifts will be defined as those with 50% more or fewer patients per nurse for each unit based on the shift median.

In the second step, the workload will be modeled with the observed-over-expected (O/E) estimator. The O/E estimator is the observed number of nurse staff on a shift divided by the expected number of nurse staff based on the shift, patient, and unit characteristics; the O/E estimator is an extension of the adjusted staffing measure we used in a previous study [23]. For the O/E estimator, we will predict the number of different staff groups (eg, RNs, nurses with ANDs, and unqualified staff) with a multivariate generalized linear model of the Poisson family, with separate models for each unit. This model will take patient characteristics; primary and secondary diagnoses; shifts; and the number of patients, admissions, transfers, and discharges into account. The observed number of nurse staff per shift will be divided by the expected value derived from the multivariate generalized linear model. The O/E estimator will then be used to describe the staffing variability over time for each patient. Furthermore, the coefficients of the model will allow us to compare units, hospitals, and countries in terms of their nurse staffing provision.

#### Aim 3

**Figure 1:**
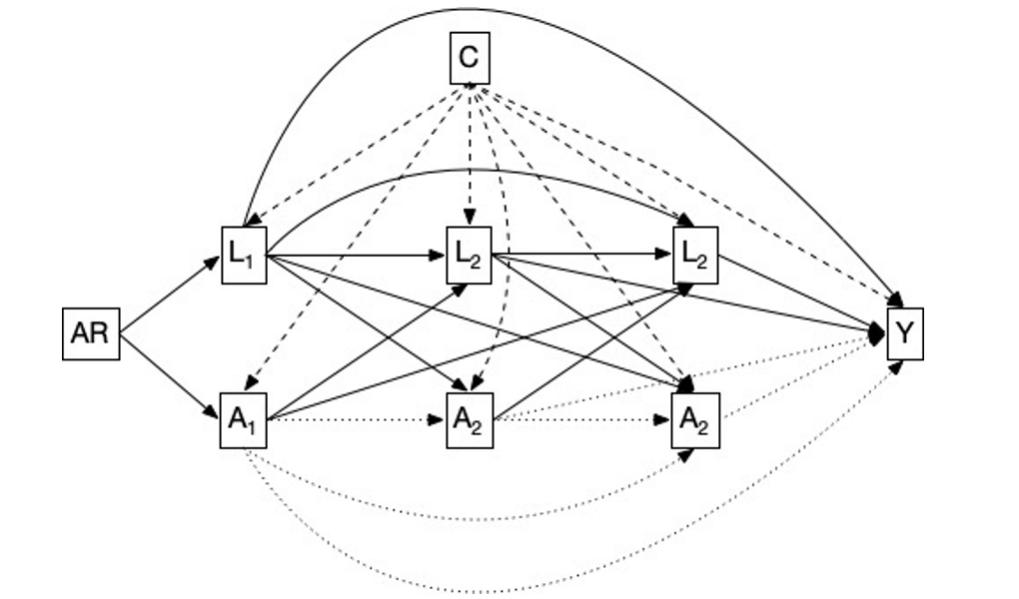
Directed acyclic graph of the staffing and nursing-sensitive events The graph describes a set of time-invariant individual-level confounders, such as age (A) or sex (C); time-variant confounders, such as patient severity on a given shift (L_1…n_); and the time-variant exposure variable of the observed-over-expected estimator for nurse staffing (A_1…n_). The minimal adjustment set also includes the admission reason (AR), which determines the unit to which a patient is admitted. Y: outcome.

We will evaluate the suspected causative effect of nurse staffing on selected NSEs using a directed acyclic graph describing the hypothesized mechanism over time (Figure 1). Directed acyclic graphs describe a set of time-invariant individual-level confounders, such as age (A) or sex (C), time-variant confounders, such as patient severity on a given shift (L_1…n_); and the time-variant exposure variable of the O/E estimator for nurse staffing (A_1…n_). The minimal adjustment set also includes the admission reason, which determines the unit to which a patient is admitted. To estimate the causal effects, 3 conditions need to be met. The first condition is the “exchangeability assumption.” An uncontrolled variable that influences exposure and outcome would violate this assumption. As the supply side (ie, the available nurse staffing resources) is determined by the unit type and relatively fixed rosters, the exposure is primarily driven by the volatility of the demand side (ie, the patients admitted to the unit). Therefore, within units, the exchangeability assumption for the exposure should hold. Indeed, occupancy might influence patient admissions or discharges, potentially creating a backdoor path. We will, therefore, consider occupancy as a variable in a sensitivity analysis. Second, the “positivity” assumption is met by the design, as individual exposure to high- or low-staffed shifts occurs continuously throughout the index admission. We will use descriptive analysis to confirm this assumption. Last, the “consistency assumption” requires that the level of exposure is identical for all patients. Although consistency is provided in the way the exposure is operationalized, the nursing staff will allocate available time between patients according to their needs within shifts. The severity measure will partially account for this. We will use nurse activity data, which are available for some sites, to explore this issue. To estimate a causal effect of nurse staffing exposure on each NSE of interest, we will construct a marginal structural model using inverse probability weighting [24]. According to VanderWeele et al [25], a marginal structural model can assess the joint effects of time-varying exposure. To calculate the weights, we will use the R package “ipw” [26]. To fit the model, we will use a generalized mixed model of the binomial family with the lme4 package in R software (version R-4.3.3; R Foundation for Statistical Computing) [27].

#### Aim 4

To help optimize and predict the necessary nursing staffing needs in hospitals to decrease NSEs, we will develop a simulation using the simmer package in R software. Simmer is a discrete-event simulation (DES) and automation software. DES is a flexible, computer-based modeling methodology characterized by the ability to simulate dynamic behaviors of complex systems and interactions between individuals, populations, and their environments. Compared with aggregate models without interaction, such as decision trees or Markov models, a DES can be more advantageous as an operational research technique to model complex systems at the individual level instead of the cohort level. After we have developed the DES model, we will test it against various data elements from our observational study, including the hospital bed size, number of nursing staff, patient flow structure, nursing practices, and NSEs. The process flow will be translated into a DES model, which will allow us to determine and validate the thresholds of safe nurse staffing levels.

### Ethical Considerations

The TAILR study is compliant with the legal and ethical guidelines of the applicable federal, clinical (including participating hospitals), and academic partners (ie, universities and universities of applied sciences). Ethical approval for the proposed study has already been obtained according to national regulations (Germany [Ethik-Kommission Hochschule für Gesundheit, University of Applied Sciences Bochum]: 210602; Sweden [Swedish Ethical Review Authority]: TAILR.SE: 2021-04962; Iran [Ethics Committee of Kashan University of Medical Sciences]: TAILR.IR: IR.KAUMS.REC.1398.032; and Switzerland [Ethikkommission Nordwest- und Zentralschweiz]: CroWiS BASEC 2022-01121).

The study will follow all ethical standards of research conduct outlined in the Declaration of Helsinki and the General Data Protection Regulation for European countries. The project team will handle the patient- and unit-level staffing data with confidentiality. Each patient record included in the record review will receive a unique identification number, and the data in a cloud-based database will be pseudonymized. Participant names will not be disclosed. The published study data will be anonymous, and therefore, there will be no identification of individual participants. The study participants received no compensation.

## Results

As of January 2024, the checking of plausibility and comprehensibility of patients' and nurse staffing data is ongoing in all 4 countries. Data analyses are planned to be completed by spring 2024, with the first results expected to be published in late 2024.

## Discussion

This paper describes the study protocol for the TAILR study. TAILR is an international multicenter study with a longitudinal and observational design that will consider within- and between-hospital variability to identify productive and urgently needed strategies to ensure safe nurse staffing levels to reduce NSEs. More concretely, TAILR will provide important building blocks to address and overcome a critical patient safety issue, that is, the lack of staffing guidance in national health policy and at local organizational levels. TAILR provides a unique opportunity to better understand the mechanisms of NSEs and the potential impact of nurse staffing on NSEs. Collecting data on nurse staffing and its association with NSEs in multiple countries, the TAILR study aims to provide a new perspective on optimal nurse staffing levels in hospitals and a unique innovative template for the evaluation of the association between NSEs in patients and individual nurse staffing levels within the international context. This study addresses a gap in current research, as emphasized by repeated calls for research to inform health policies on safe nurse staffing levels [15,28]. This need has been increased due to the COVID-19 pandemic and the increasing pressures to develop and maintain the nursing workforce in the participating countries as well as other countries [29].

TAILR will act as a model for nurse-led improvement strategies for safe staffing within different countries. First, for the participating hospitals, TAILR will create transparency by providing comparative data. Moreover, we will be able to identify hospital structures and processes that facilitate and challenge safe nurse staffing. By conducting workshops with the participating hospitals and stakeholders, the best practices will be identified and described to create applicable solutions based on the study results. Second, based on the workshops with hospitals and stakeholders, TAILR will provide data to develop recommendations that provide cornerstones for safe staffing policies in a country-specific context. Beyond mandatory minimum nurse staffing levels, TAILR will provide highly granular data to describe operational gaps in nurse staffing, which remain undetected in aggregate analysis. Potential safe staffing policies must consider the service line and patient acuity as well as hospital-specific factors, such as nurse pools, clear distribution of nursing and nonnursing tasks between nurses with different educational backgrounds, and the systematic assessment and monitoring of nurse staffing and NSEs. Third, the international TAILR study will create research outputs for the participating countries as well as other countries. For (inter)national stakeholders and the members of the international TAILR consortium, the study results will enable the definition of global quality improvement strategies.

## Appendix

Multimedia Appendix 1 Peer-review report by the German Federal Ministry of Education and Research.

## Abbreviations

AND: associate nursing degree
CroWiS: Crowd Working in der Schweiz
DES: discrete-event simulation
ICC: intraclass correlation coefficient
NSE: nursing-sensitive event
O/E: observed-over-expected
RN: registered nurse
RRR: retrospective record review
TAILR: Nursing-Sensitive Events and Their Association With Individual Nurse Staffing Levels

## Data Availability

The data that support the findings of this study are available from the corresponding authors, (SB, MU, and MS) upon reasonable request.

## References

[1] D'Amour D, Dubois C, Clarke S, Blais R, Tchouaket (2014). The occurrence of adverse events potentially attributable to nursing care in medical units: cross sectional record review. Int J Nurs Stud.

[2] Grube J (2004). National voluntary consensus standards for hospital care: an initial performance measure set. JHQ.

[3] Needleman J, Buerhaus P, Mattke S, Stewart M, Zelevinsky K (2002). Nurse-staffing levels and the quality of care in hospitals. N Engl J Med.

[4] Griffin FA, Resar RK (2009). IHI Global Trigger Tool for Measuring Adverse Events (Second Edition).

[5] Hommel A, Magnéli M, Samuelsson B, Schildmeijer K, Sjöstrand D, Göransson KE, Unbeck M (2020). Exploring the incidence and nature of nursing-sensitive orthopaedic adverse events: a multicenter cohort study using Global Trigger Tool. Int J Nurs Stud.

[6] Nilsson L, Lindblad M, Johansson N, Säfström L, Schildmeijer K, Ekstedt M, Unbeck M (2023). Exploring nursing-sensitive events in home healthcare: a national multicenter cohort study using a trigger tool. Int J Nurs Stud.

[7] Murphy A, Griffiths P, Duffield C, Brady NM, Scott AP, Ball J, Drennan J (2021). Estimating the economic cost of nurse sensitive adverse events amongst patients in medical and surgical settings. J Adv Nurs.

[8] Bridges J, Griffiths P, Oliver E, Pickering RM (2019). Hospital nurse staffing and staff-patient interactions: an observational study. BMJ Qual Saf.

[9] Griffiths P (2014). The association between patient safety outcomes and nurse/healthcare assistant skill mix and staffing levels and factors that may influence staffing requirements. University of Southampton.

[10] Needleman J, Buerhaus P, Pankratz VS, Leibson CL, Stevens SR, Harris M (2011). Nurse staffing and inpatient hospital mortality. N Engl J Med.

[11] Cho E, Chin DL, Kim S, Hong O (2016). The relationships of nurse staffing level and work environment with patient adverse events. J Nurs Scholarsh.

[12] Aiken LH, Sloane DM, Bruyneel L, Van den Heede K, Griffiths P, Busse R, Diomidous M, Kinnunen J, Kózka M, Lesaffre E, McHugh MD, Moreno-Casbas MT, Rafferty AM, Schwendimann R, Scott PA, Tishelman C, van Achterberg T, Sermeus W (2014). Nurse staffing and education and hospital mortality in nine European countries: a retrospective observational study. The Lancet.

[13] Aiken LH, Clarke SP, Sloane DM, Lake ET, Cheney T (2009). Effects of hospital care environment on patient mortality and nurse outcomes. J Nurs Adm.

[14] Dall'Ora C, Saville C, Rubbo B, Turner L, Jones J, Griffiths P (2022). Nurse staffing levels and patient outcomes: a systematic review of longitudinal studies. Int J Nurs Stud.

[15] Needleman J, Shekelle PG (2019). More ward nursing staff improves inpatient outcomes, but how much is enough?. BMJ Qual Saf.

[16] Griffiths P, Ball J, Drennan J, Dall'Ora C, Jones J, Maruotti A, Pope C, Recio Saucedo A, Simon M (2016). Nurse staffing and patient outcomes: strengths and limitations of the evidence to inform policy and practice. A review and discussion paper based on evidence reviewed for the National Institute for Health and Care Excellence Safe Staffing guideline development. Int J Nurs Stud.

[17] Musy SN, Endrich O, Leichtle AB, Griffiths P, Nakas CT, Simon M (2020). Longitudinal study of the variation in patient turnover and patient-to-nurse ratio: descriptive analysis of a swiss university hospital. J Med Internet Res.

[18] Griffiths P, Maruotti A, Recio Saucedo A, Redfern OC, Ball JE, Briggs J, Dall'Ora C, Schmidt PE, Smith GB, Missed Care Study Group (2019). Nurse staffing, nursing assistants and hospital mortality: retrospective longitudinal cohort study. BMJ Qual Saf.

[19] CroWiS.

[20] Blume K, Dietermann K, Kirchner-Heklau U, Winter V, Fleischer S, Kreidl LM, Meyer Gabriele, Schreyögg Jonas (2021). Staffing levels and nursing-sensitive patient outcomes: Umbrella review and qualitative study. Health Serv Res.

[21] Jones PS, Lee JW, Phillips LR, Zhang XE, Jaceldo KB (2001). An adaptation of Brislin's translation model for cross-cultural research. Nurs Res.

[22] Austin PC (2021). Informing power and sample size calculations when using inverse probability of treatment weighting using the propensity score. Stat Med.

[23] Bachnick S, Ausserhofer D, Baernholdt M, Simon M, Match RN study group (2018). Patient-centered care, nurse work environment and implicit rationing of nursing care in Swiss acute care hospitals: a cross-sectional multi-center study. Int J Nurs Stud.

[24] Robins JM, Hernán MA, Brumback B (2000). Marginal structural models and causal inference in epidemiology. Epidemiology.

[25] VanderWeele TJ, Jackson JW, Li S (2016). Causal inference and longitudinal data: a case study of religion and mental health. Soc Psychiatry Psychiatr Epidemiol.

[26] van der Wal WM, Geskus RB (2011). ipw: an R package for inverse probability weighting. J Stat Soft.

[27] Halekoh U, Højsgaard S, Yan J (2006). The package for generalized estimating equations. J Stat Soft.

[28] Butler M (2019). Hospital nurse-staffing models and patient- and staff-related outcomes. Cochrane Database Syst Rev.

[29] Googe MC (1982). Nurse retention. Orthop Nurs.

