# Supplementary material for: TAILR (Nursing-Sensitive Events and Their Association With Individual Nurse Staffing Levels) Project: Protocol for an International Longitudinal Multicenter Study
Source: JMIR Res Protoc. 2024 Apr 22;13:e56262. doi: 10.2196/56262 (PMC11074892; doi:10.2196/56262)
Supplement: Multimedia Appendix 1 [file resprot_v13i1e56262_app1.pdf]

**Nurse-sensiTive events in older patients and the Association with Individual patient Levels nuRse staffing in German hospitals (TAILR.DE)**		**PFLEGE_M2_NWG-001**
Junior Research Project Leader:	Dr. Stefanie Bachnick	
Institution:	Hochschule für Gesundheit (hsg), University of Applied Sciences; Department of Nursing Science; Department of Nursing Science	
Requested funding:		

Summary: Nursing sensitive events (NSEs) are unintended physical injury affected, delivered or influenced by nursing care. The main goal of the TAILR.DE study is to describe the association of NSEs in older patients and individual level of nurse staffing in German hospitals. Specific aims are: 1) To determine the frequency, severity, preventability and types of NSEs across sites with a structured record review methodology; 2) To describe individual patient-level nurse staffing in medical-surgical patients at multiple sites; 3) To describe the association between NSEs and nurse staffing in medical-surgical patients; 4) To determine thresholds of safe nurse staffing levels and test them against NSEs across sites. TAILR.DE is a 3-year multi-center retrospective longitudinal observational study using a participatory research design. From 3 hospitals, 4 units each will be included to collect patients' data (i.e., NSEs with standardised retrospective chart reviews) and nurses-staffing shift data (standardises shift-level staffing template) over a period of 16 weeks.

Evaluation: It is an asset of the proposed study to be embedded in the international TAILR project. TAILR will provide data on NSEs (nurse sensitive events) in older patients and individual nurse staffing levels within the international/German context, comprehensive data collection, variety of settings included. The proposed study topic is at high priority in nursing science and this study has innovative study questions as well as very well planned design and methods. Minor weaknesses of the proposal are that the outcomes are not clearly defined and that a clear plan for statistical analysis as well as a formal power analysis to determine the targeted sample size for the NSE detection are lacking.
The proposed study bases on the former PhD study of the junior researcher (PI), which will be further developed. The PI has a clear independent role as a nursing science researcher and is embedded into excellent national and international academic, health service and policy collaboration. The project contributes to the research profile of the junior project leader and the institution.
All in all, the described work plan seems to be realistic. However, some parts need minor revision, see below. The funds applied for appear adequate.

The following advice is to be taken into account for the submission of the formal appropriation request (*Formantrag*):

- Given the complexity of the variables considered herein (e.g. patient characteristics and comorbidity, hospital and unit-related variables) and the potential interrelations between them, a well-elaborated and detailed analysis plan is needed. The analysis needs to include extensive controls for potential sources of an increased risk of events other than nurse staffing. Please provide an analysis plan.
- The primary endpoints include NSEs; however, it is not clear which outcomes will be assessed. Please comment and add detailed information on the outcome that may benefit evaluation of the proposal and also the statistical analyses.
- How will you react to changes in unit- and patient-level variables in your analysis?

- How do you take into account important differences between the units (surgical, medical or mixed)?
- Although it is stated that many aspects (e.g. patient turnover, educational background of the nurses) need to be taken into account, it is not clear whether these variables are addressed in the proposed study. Please comment.
- Elaborate in detail on the statistical expertise involved into your project.
- Reconsider your work plan. The timeframe for data collection and analysis appears to be tight.

Recommendation: Recommended for funding with recommendations (minor revisions)

Funds recommended
(Subject to further revision of allocation and eligibility by the DLR-PT)
